# Pulp response of rats submitted to bleaching and the use of different anti-inflammatory drugs

**DOI:** 10.1371/journal.pone.0210338

**Published:** 2019-01-08

**Authors:** Marjorie de Oliveira Gallinari, Luciano Tavares Ângelo Cintra, Francine Benetti, Vanessa Rahal, Edilson Ervolino, André Luiz Fraga Briso

**Affiliations:** 1 Department of Restorative Dentistry, Araçatuba Dental School, UNESP—Univ Estadual Paulista, Araçatuba, São Paulo, Brazil; 2 Department of Basic Sciences, Araçatuba Dental School, UNESP—Univ Estadual Paulista, Araçatuba, São Paulo, Brazil; Medical University of Vienna, AUSTRIA

## Abstract

This study aimed to evaluate neuropeptide expression after bleaching treatment using histopathological and immunohistochemical analyses and the effects of hydrocortisone and acetaminophen on pulp inflammation, sine dental bleaching and inflammation first occur, and only then, the treatmentt. Sixty-three rats were divided into three groups (n = 21) according to the pain-relieving therapy used: I-control; II-topical application of Otosporin for 10 min after the bleaching treatment; III-oral administration of paracetamol 30 min before whitening and then every 12h. In all the study groups, placebo gel was applied to the left upper jaw (control) and a 35% H_2_O_2_-based whitening gel was applied to the right upper jaw for 45 min. Seven animals from each group were euthanized at different time points: 0h after treatment, 24h, and 48h. After euthanasia, the first molar on each side was analyzed by histology and immunohistochemistry to assess the degree of inflammation and verify the presence of the neuropeptides, substance P (SP) and calcitonin gene-related peptide (CGRP). The data were analyzed using the statistical nonparametric Kruskal-Wallis test followed by Dunn's test for individual comparisons. Extensive areas of necrosis were observed in the groups that received bleaching treatment only, whereas reduced damage were obtained in the group treated with Otosporin. The immunohistochemical analysis showed positive immunolabeling in all groups, including the control, but this was stronger in the groups that received bleaching treatment. The best results were obtained in the group that received treatment with Otosporin. The use of Otosporin after dental bleaching minimized the side effects of this treatment.

## Introduction

Demand for esthetic procedures has increased in contemporary dentistry and dental bleaching is one of the clinical procedures most frequently requested by patients. This treatment can be performed at home by daily exposure to low-concentration peroxides or in office treatment, by using highly concentrated peroxides. Both techniques are based on the release of reactive oxygen species (ROS), which are extremely unstable and cleave the chromophores present in the dental structure, transforming them into smaller molecules and thereby making the teeth whiter [1–6]. Despite the esthetic improvement, the penetration of HP and its toxic by products into the pulp-dentin complex [7–9] is responsible for pulpar damage ranging from a transient inflammatory response to the occurrence of local necrosis [10–12].

However, the action of ROS is not limited to the oxidation of pigment substances, and there have been reports of significant concentrations of peroxide in the pulp chamber after application of the bleaching gel to the dental enamel [13]. This fact has been associated with morphological changes decreased mitochondrial respiration rates in MDPC-23 odontoblast cells [13], increase of dental hypersensitivity and irreversible damage to the pulp [14]. *In vivo* studies in rats have confirmed the presence of damage to the pulp cells in animals that received bleaching treatment, and that such damages were proportional to the number of bleaching sessions [11].

Cellular damage caused by the penetration of hydrogen peroxide (H_2_O_2_) induces the synthesis and release of biochemical mediators, such as prostaglandins, histamine, and bradykinin, that are involved in the inflammatory process [11, 13]. These mediators cause an increase in vascular permeability and vasodilation within the pulp cavity [13]. Any increase in pulp pressure mechanically stimulates the peripheral nerve fibers [15], which respond with the production and release of peptide neurotransmitters, including substance P (SP) and calcitonin gene-related peptide (CGRP) [15, 16]. These neuropeptides excite transmission neurons, thereby promoting the emission of pain signals from the area of tissue injury [17]. This phenomenon is the cause of frequent reports of discomfort and pain in patients undergoing bleaching treatment [4].

However, sensitivity is most often assessed subjectively, making it difficult to investigate and compare the efficacy of different pain relief methods. Therefore, monitoring SP and CGRP levels can provide objective insight into inflammation and pain during bleaching treatment.

In clinical practice, oral and topical drugs are administered to minimize the clinical effects of these inflammatory mediators, and desensitizing agents are used before or after the bleaching procedure [18]. These desensitizing agents may act by sealing the dentinal tubules (physical action) and/or by blocking the nervous stimulus (neural action) [19].

Hydrocortisone is a topical anti-inflammatory steroidal, commercially available under the name of Otosporin and often used in combination with the antibiotic neomycin sulfate. It has been used in dentistry as an endodontic medicine, and for the treatment of dentin hypersensitivity after restorations, to attenuate the intensity of the inflammatory reaction, eliminate postoperative pain, and promote tissue repair [20, 21]

Acetaminophen is an analgesic and antipyretic medication that inhibits the arachidonic acid cascade, preventing the synthesis of prostaglandins and reducing vascular permeability and pain [22]. Acetaminophen inhibits a variant of the enzyme cyclooxygenase (COX) [23] and is therefore an excellent pain-relieving drug with few side effects.

These and other drugs may control tooth sensitivity, but they do not prevent pulp damage. To promote a tissue repair and reduce the dental sensitivity resulting from the bleaching treatment, it is important to study the effects of some pain-relieving therapies on pulp inflammation and on the expression of the neurotransmitters SP and CGRP in the pulp tissue. This information may contribute to the determination of new parameters for dental bleaching, aiming at the development of more efficient protocols, with minimal side effects related to tooth sensitivity and pulpal alterations. Therefore, the aim of this study was to assess the effects of pain-relieving therapies on inflammation and expression of pro-inflammatory neuropeptides after bleaching treatment performed with high-concentration peroxide.

## Materials and methods

Sixty-three male rats (*Rattus albinus*, Wistar) weighing approximately 200–250 g were used in this study, totalazing 126 jaws (right and left). The animals were kept in an air-conditioned environment, with a temperature between 22 and 24°C, controlled light cycles (12 light hours and 12 dark hours), and *ad libitum* access to water and food.

This study was carried out in strict accordance with the recommendations in the Guide for the Care and Use of Laboratory Animals of the National Institutes of Health (Bethesda, MD, USA). The protocol was approved by the local ethics committee, the Ethics Committee on the Use of Animals of the Araçatuba Dental School, UNESP-Univ Estadual Paulista (Protocol Number: 2014–00817). All surgery was performed under sodium pentobarbital anesthesia, and all efforts were made to minimize suffering.

The rats were randomly assigned to three lot according to the treatment used for pain control (21 rats per lot):

Ctrl–No drug was used in this lot

Oto–The animals in this lot received the topical application of Otosporin (Hydrocortisone, neomycin sulfate and polymyxin B sulfate, Farmoquímica S/A, Rio de Janeiro, RJ, Brazil) after the bleaching treatment. Therefore, 3 drops of Otosporin were applied in the upper jaw area for 10 minutes on both sides, left and right molars using hydrophilic cotton (Table 1).

Tyl–The animals in this lot received Tylenol 40mg/ml/kg [24] (Paracetamol, Janssen-Cilag Pharmaceuticals Ltda, São Paulo, SP, Brazil). A 500 mg tablet of the drug Paracetamol was diluted in 100 ml of distilled water and administered orally [24]. This solution was given 30 min before the start of the bleaching treatment, then once every 12 h until euthanasia (Table 1).

**Table 1:** Distribution of the experimental groups according to treatment received, ethanasia times, and treatment location.

Groups	Analysis times	Maxillary left	Maxillary right
I Control	0 hour (n = 7)	G1—Placebo gel	G2—Hydrogen peroxide 35%*
	24 hours (n = 7)		
	48 hours (n = 7)		
II Otosporin	0 hour (n = 7)	G3—Placebo gel + Otosporin	G4—Hydrogen peroxide 35%* + Otosporin **
	24 hours (n = 7)		
	48 hours (n = 7)		
III Tylenol	0 hour (n = 7)	G5—Placebo gel + Tylenol	G6—Hydrogen peroxide 35%* + Tylenol ^†^
	24 hours (n = 7)		
	48 hours (n = 7)		

*FGM Produtos Odontológicos, Santa Catarina, Brazil

** Laboratório Farmoquímica, Rio de Janeiro, Brazil

^†^ Janssen-cilag Farmacêutica Ltda., São Paulo, Brazil

**Fig 1:**
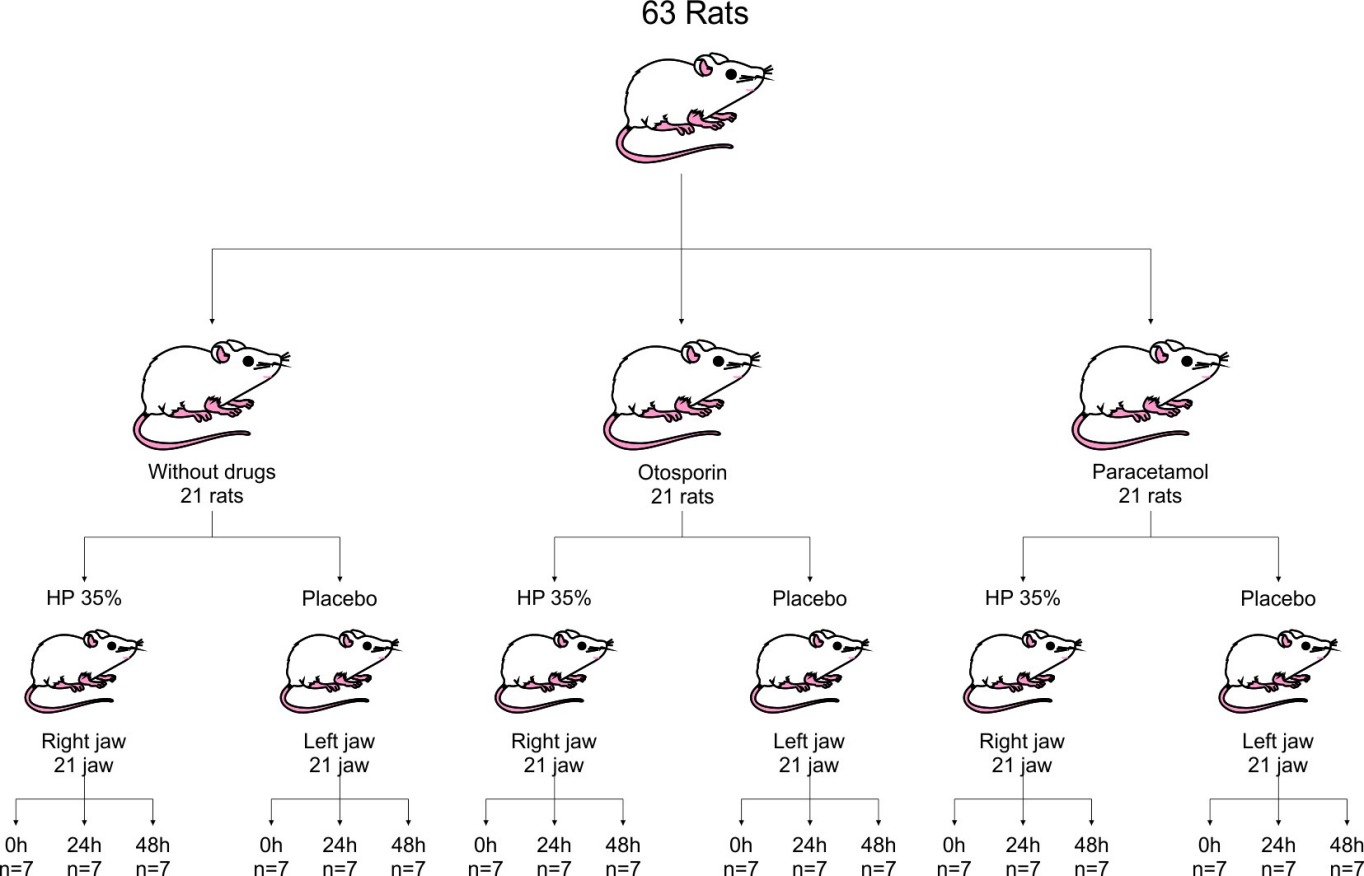
Flowchart detailing the division of animals in the sutudy groups.

Each lot was divided into two groups according to the bleaching gel used (21 jaws per group), placebo or hydrogen peroxide. These groups were then subdivided according to the time of analysis: immediate, 24 and 48 hours (n = 7) (Fig 1).

Thereby, a split mouth design was established after the bleaching procedure. The placebo gel (Pla) was applied to the left maxilla and the 35% hydrogen peroxide (Bleach) in the right maxilla, resulting in six groups: control (PlaCtrl), bleaching treatment control (Bleach), Otosporin control (PlaOto), bleaching treatment with Otosporin (BleachOto), Tylenol control (PlaTyl), and bleaching treatment with Tylenol (BleachTyl) (Fig 1).

In order to perform the bleaching treatment, the animals in groups Ctrl and Oto were given intramuscular injections of the sedatives xylazine hydrochloride (Dopaser, Calier SA, Barcelona, Spain) at a dose of 13 mg/kg and ketamine hydrochloride (Vetanarcol, König SA—Avellaneda, Argentina) at a dose of 25 mg/kg for anesthesia. Group Tyl animals received the sedative 30 minutes after administration of the pain-relieving drug. After anesthesia, 1 μL of gel-based 35% H_2_O_2_ (Whiteness HP Maxx; FGM Produtos Odontológicos, Joinville, SC, Brazil) was applied to the upper right molars, and remainedin place for 45 minutes before washing off. The upper left molars were treated in the same manner with placebo gel (controls), product with the same clinical presentation (color and viscosity) of the in-office bleaching gel used in the right jaw, but without the active principle (hydrogen peroxide) (Table 1).

Seven animals in each group were euthanized at different time points: immediately after treatment, after 24 h, or after 48 h. For euthanasia, the animals were anesthetized as mentioned previously and were subjected to transcardiac perfusion, starting with 100 mL 0.9% sodium chloride solution, followed by 500 mL fixative solution consisting of 4% formaldehyde (Sigma-Aldrich, MO, United States) and 3.8% sodium tetraborate (Sigma-Aldrich, MO, United States) at 0.1 M, 4°C, and pH 9.5. The jaws were then dissected.

The tissues were kept for 24 h in formaldehyde, and then decalcified in 10% EDTA (Ethylenediaminetetraacetic acid,Sigma-Aldrich, MO, United States) for 90 days. The tissues were processed in the conventional manner and embedded in paraffin^6^. Twelve cuts (6 μm) were obtained from the mesial plane of each specimen, eight of which were used for hematoxylin-eosin (HE) analysis and the other four for immunohistochemistry (IH) analysis.

Histological preparations intended for HE analysis were observed under optical microscope (Leica Microsystems—DM 4000 B, Wetzlar, Germany) at 400x magnification. The pulp tissue sections stained with HE were scored according to the presence of inflammatory infiltrate as follows: 1—absence of inflammatory cells or negligible number thereof; 2—minimal inflammatory infiltrate; 3—moderate inflammatory infiltrate; 4—severe inflammatory infiltrate; 5—necrosis and absence of any cell type (modified from Cintra *et al*., 2013) [11].

For immunohistochemical reactions, the histological slides were deparaffinised (xylene) and hydrated (decreasing ethanol series). Antigen retrieval was achieved by immersing the histological slides in buffer citrate solution (Antigen Retrieval Buffer, Spring Bioscience, Pleasanton, CA, USA) in a pressurised chamber (Decloaking Chamber; Biocare Medical, Concord, CA, USA) at 95°C for 10 minutes. The slides were rinsed with phosphate-buffered saline (PBS) at the end of each stage of the immunohistochemical reaction. The histological slides were immersed in 3% H_2_O_2_ solution (1 h and 20 min) to block endogenous peroxidase activity, and in 1% bovine serum albumin (12 h) to block the nonspecific sites.

After, the slides were divided according to the markings to be performed with the SP and CGRP neuropeptides. Subsequently, one of the following primary antibodies was incubated for 24 h n: rabbit anti-SP (AB1566, Millipore, Darmstadt, Germany) or rabbit anti-CGRP (AB91007, ABCAM Plc, Cambridge, United Kingdom). Primary antibodies were diluted in Dako Antibody Diluent (Dako Laboratories, CA, USA) in the 1:250 ratio. In subsequent steps the Universal Dako Labeled (HRP) Streptavidin-Biotin Kit (Dako Laboratories, CA, USA) was used. Histological sections were incubated in the biotinylated secondary antibody for 2 hours and treated with streptavidin conjugated with the horseradish peroxidase (HRP) for 1 hour. In the disclosure, diaminobenzidine 3,3'-tetrahydrochloride (DAB chromogen Kit, Dako Laboratories, CA, USA) was used as the chromogen. All groups were submitted to the immunohistochemical reactions at the same time so that there were no variations of the test, which could interfere in the semi-quantitative analyzes (score).

As a positive control of immunostaining, the protocol described above was used in samples from the rat trigeminal ganglion, which shows pericaries of neurons and nerve fibers immunoreactive to SP and CGRP. As negative control of the immunohistochemical reaction, histological sections were used where the above protocol was used, however, with the suppression of the primary antibodies.

Treatment groups were blinded to the observer. Positive immunolabeling was defined as the presence of a brown color in cells, nerve fibers, and the extracellular matrix. For each marker, four histological sections equidistant from the dental pulp of the first maxillary molar were used. The immunohistochemical analysis was performed on thirds of the coronal pulp (occlusal, middle, and cervical sections) and radicular pulp (coronal, middle, and apical). Scores were assigned as follows: 1—absence of immunostaining; 2—minimal immunolabeling pattern; 3—average immunolabeling pattern; 4—high immunolabeling pattern; 5—necrotic cell remains.

### Statistical analysis

The Statistical Package (Pacotico) software was used for statistical analysis. The normality and the homoscedasticity of the data were analyzed and the nonparametric Kruskal-Wallis test was performed, followed by Dunn’s test, for individual comparisons. The level of significance was 5% for all tests.

## Results

### Analysis of controls groups

Statistical analyses of the control groups (PlaCtrl, PlaOto, and PlaTyl), for both the histological and immunohistochemical analyses, were performed independently from the treatment groups to analyze the isolated effects of the drugs on pulp tissue health. Normal tissue was observed and similar biological responses were observed in all groups that received placebo gel, indicating that no damage to the pulp tissue occurred after treatment with placebo gel or the drugs tested.

The IH analysis showed light immunolabeling for both neuropeptides (SP and CGRP) in the three control groups (PlaCtrl, PlaOto, and PlaTyl) in all sections and time points analyzed. There were no statistically significant differences.

### Histological analysis

**Fig 2:**
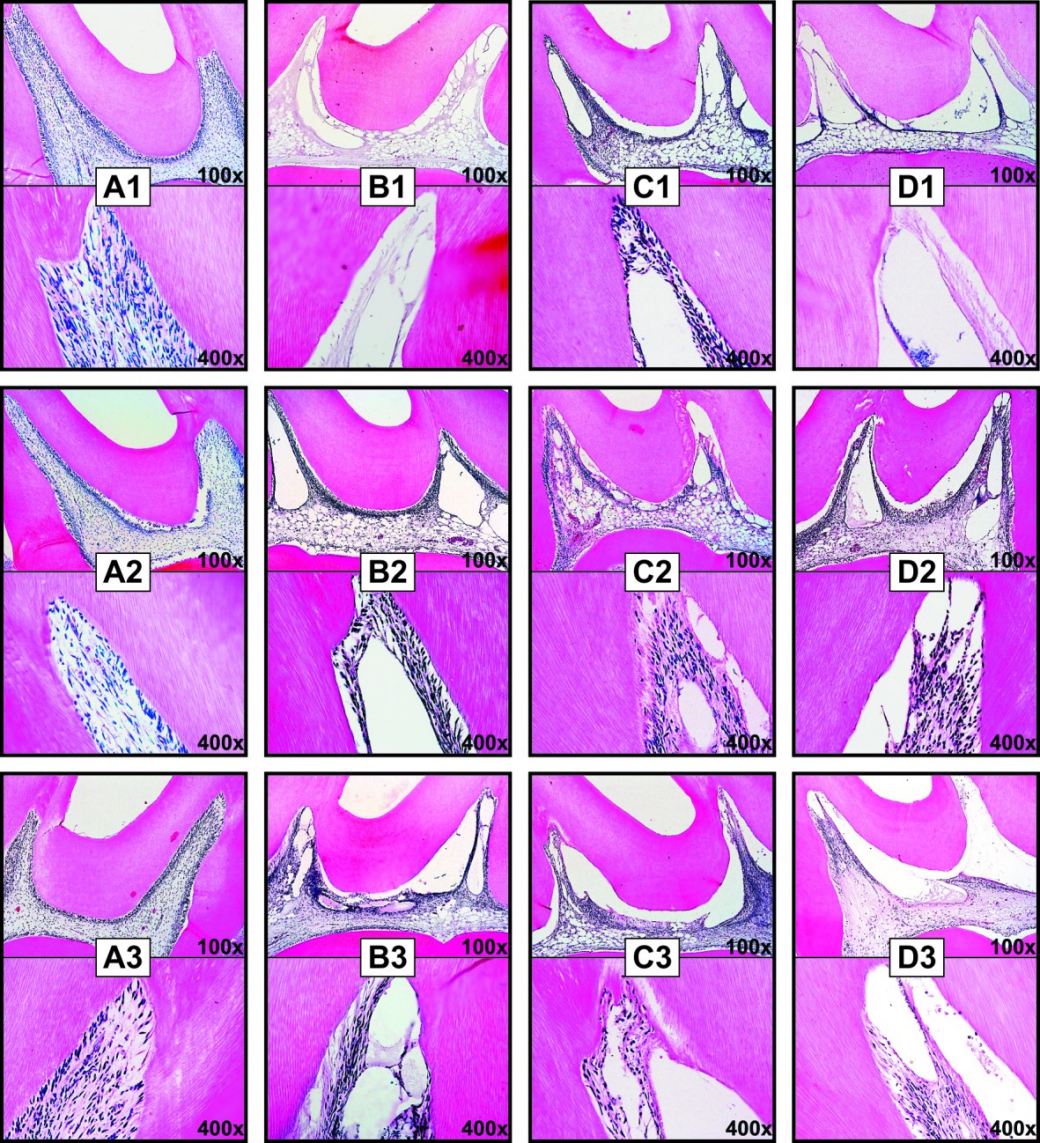
Longitudinal section of the coronal thirds of the pulp tissue, at the first time point (1), 24 h after bleaching (2), and 48 h after bleaching (3). **Samples were stained with HE and observed under an optical microscope (400x).** *(A1-D1) Immediately after bleaching: Representative photomicrographs of the control group (A1) show normal pulp tissue and intact odontoblastic layer (black arrows), and evidenced tissue organization; the Bleach (B1) and BleachTyl group (D1) shows areas of necrosis and a decrease in cellularity in the occlusal third, (c2) that were evidenced in higher magnification; and the BleachOto group (C1) presents cellular disorganization (black arrowheads) and (b2) severe inflammatory infiltrate (yellow arrowheads) in the occlusalt hird. (A2-D2) At 24h after bleaching: Representative photomicrographs of the Bleach group (B2) and BleachTyl (D2) presents severe inflammatory infiltrate (yellow arrowheads) and BleachOto group (C2) showing moderate inflammatory infiltrate (red arrowheads) in the occlusalt hird. At 48h after bleaching: Representative photomicrographs of the Bleach group (B3) and BleachTyl (D3) presents moderate inflammatory infiltrate (red arrowheads) and BleachOto group (C3) showing absence of inflammatory infiltrate (blue arrowheads) in the occlusalt hird. [H&E staining, 100x].

The scores assigned to each group can be observed in Table 2 and Fig 2. At 0 hours, there was necrosis in most specimens in occlusal third of the Bleach and BleachTyl groups (p>0.05); in the BleachOto group, there was moderate inflammatory infiltrate similary to the Bleach group. However, despite the alterations previously reported and observed in Fig 2, the samples from the BleachOto group was statistically similar to the ones from the PlaCtrl group (Fig 2). At 24 h and 48 h time points, all specimens had cellular disorganization, especially inthe pulp horns; most specimens had moderate inflammation in this region, whereas in the middle and cervical third, mild inflammation was observed (Fig 2).

**Table 2:** Median scores assigned in the histological analysis of each coronal and radicular third, for all bleached groups.

		*Ctrl*			*Bleach*			*BleachOto*			*BleachTyl*		
Thirds		0h	24h	48h	0h	24h	48h	0h	24h	48h	0h	24h	48h
**Crown**	*Occlusal*	1 Ab	1 Ab	1 Ab	5 Aa	4 Ba	4 Ba	3 Bab	3 Bab	1 Ab	5 Aa	4 Ba	4 Ba
	*Medium*	1 Ab	1 Ab	1 Ab	5 Aa	3 Ba	3 Ba	2 Bab	2 Bab	1 Ab	4 Aa	3 ABa	3 Ba
	*Cervical*	1 Ab	1 Ab	1 Ab	5 Aa	2 Ba	2 Ba	2 Bab	2 Ba	1 Ab	4 Aa	2 Ba	2 Ba
**Root**	*Coronáry*	1 Ab	1 Aa	1 Aa	4 Aa	1 Ba	1 Ba	2 Aab	1 Ba	1 Ba	3 Aa	1 Ba	1 Ba
	*Medium*	1 Ab	1 Aa	1 Aa	2 Aa	1 Ba	1 Ba	1 Ab	1 Aa	1 Aa	2 Aa	1 Ba	1 Ba
	*Apical*	1 Aa	1 Aa	1 Aa	1 Aa	1 Aa	1 Aa	1 Aa	1 Aa	1 Aa	1 Aa	1 Aa	1 Aa

* Means followed by different letters represent significant difference according to statistical analysis (p,0.05). Uppercase mean comparison between times within a group, and lowercase mean comparison between groups within a time (0h, 24h or 48h).

### Immunohistochemical analysis

#### Immunolabeling SP of experimental groups

**Fig 3:**
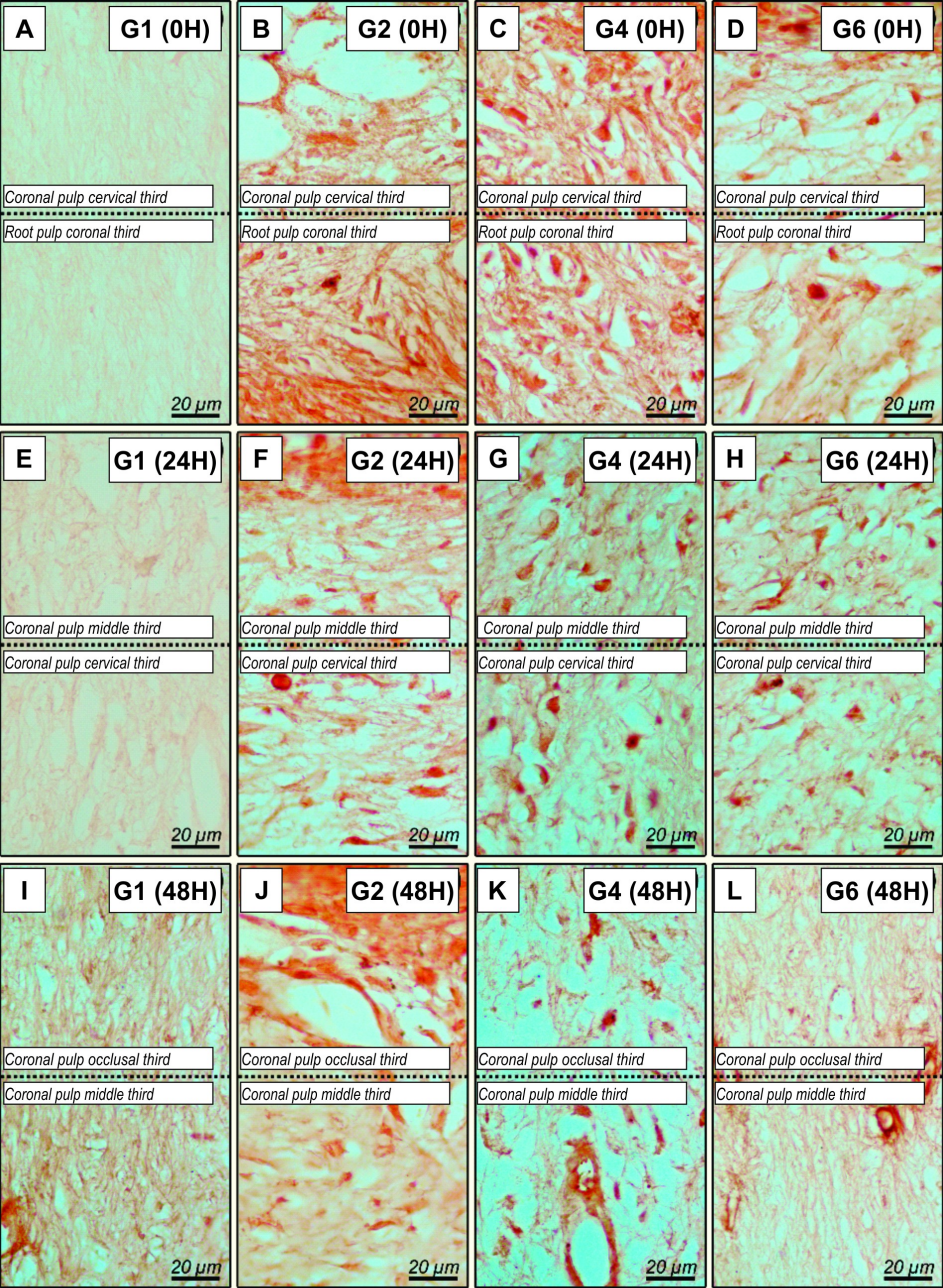
Histological sections showing immunolabeling patterns for SP. * Representative photomicrographs of the immunolabelling for SP at 0h (A-D) in the control group showing low immunolabelling (A), Bleach group showing areas of necrosis (B), BleachOto group and (C) and BleachTyl (D) showing moderate immunolabelling; and 24h control (E), BleahOto (G) and BleachTyl (H) showing low immunolabelling, and Bleach group (F) showing high immunolabelling; at 48h control (I), BleachOto (K) and BleachTyl (L) showing low immunolabelling, and Bleach group (F) showing moderate immunolabelling. Original magnification: 1000x.

The representative images of immunolabelling for SP can be visualized in Fig 3. At 0h, SP analysis revealed that the PlaCtrl group had low immunolabelling (Table 3 and Fig 3A); the Bleach presented areas of necrosis in all coronal sections analyzed (Fig 3B). BleachOto and BleachTyl exhibited high immunolabelling (Fig 3C and 3D). At 24 h only the Bleach group presented high immunolabelling (Fig 3F and 3G). At 48 h (Fig 3I–3L), BleachOto and BleachTyl groups had mild immunolabelling for SP. The analysis according to time, it was observed that Bleach group showed a decrease in marking, whereas PlaCtrl, BleachOto, and BleachTyl maintained their characteristics from the 24 hour analysis time.

**Table 3:** Median scores assigned to SP immunolabeling in each coronal third for all groups studied.

		*Ctrl*			*Bleach*			*BleachOto*			*BleachTyl*		
Thirds		0h	24h	48h	0h	24h	48h	0h	24h	48h	0h	24h	48h
**Crown**	*Occlusal*	2Ab	2Ab	2Ab	5Aa	4ABa	3Ba	4Aab	3ABab	2Bb	5Aab	3ABab	2Bb
	*Medium*	2Ab	2Ab	2Ab	5Aa	4ABa	2Ba	4Aab	2Bb	2Bb	4Aab	3ABab	2Bb
	*Cervical*	2Ab	2Ab	2Ab	5Aa	3ABa	2Bb	3Ab	2Bab	2Bb	4Aab	2Bab	2Bb
**Root**	*Coronary*	2Ab	2Ab	2Aa	4Aa	3ABa	3Ba	3Ab	2Ab	2Aa	2Ab	2Aab	2Aa
	*Medium*	2Ab	2Aa	2Aa	4Aa	2Ba	2Ba	2Ab	2Aa	2Aa	2Ab	2Aa	2Aa
	*Apical*	2Aa	2Aa	2Aa	2Aa	2Aa	2Aa	2Aa	2Aa	2Aa	2Aa	2Aa	2Aa

* Means followed by different letters represent significant difference according to statistical analysis (p,0.05). Uppercase mean comparison between times within a group, and lowercase mean comparison between groups within a time (0h, 24h or 48h).

#### Immunolabeling CGRP of experimental groups

**Fig 4:**
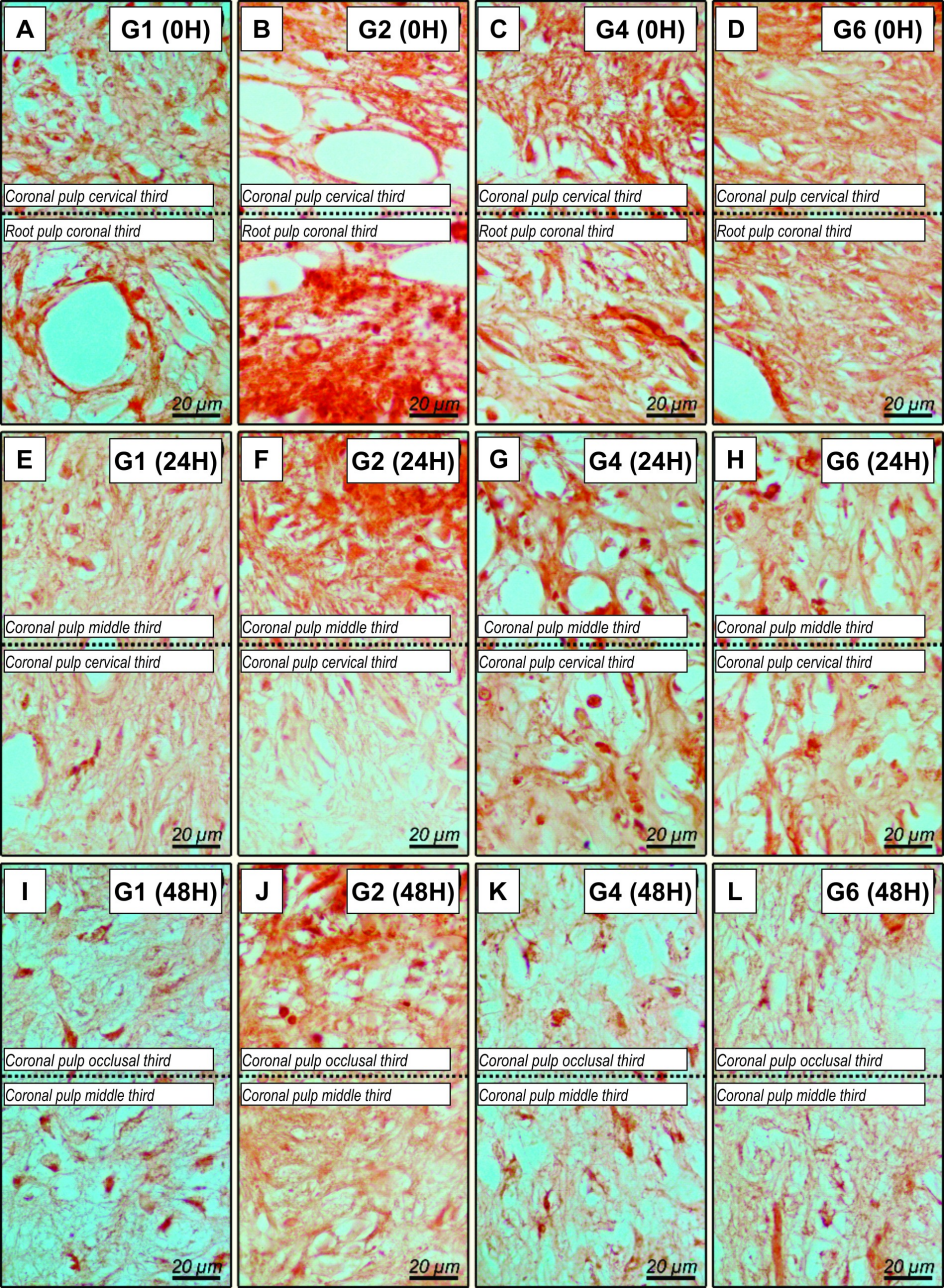
Histological sections showing immunolabeling patterns for CGRP. * Representative photomicrographs of the immunolabelling for SP at 0h (A-D) in the control group showing low immunolabelling (A), Bleach group showing areas of necrosis (B), BleachOto group and (C) and BleachTyl (D) showing moderate immunolabelling; and 24h control (E) showing low immunolabelling, BleahOto (G) and BleachTyl (H) showing moderate immunolabelling, and Bleach group (F) showing high immunolabelling; at 48h control (I), BleachOto (K) and BleachTyl (L) showing low immunolabelling, and Bleach group (F) showing moderate immunolabelling. Original magnification: 1000x.

The representative images of immunolabelling for CGRP can be visualized in Fig 4 and Table 4. At 0h, CGRP analysis revealed that the PlaCtrl group had low immunolabelling (Fig 4A); the Bleach presented areas of necrosis in all coronal sections analyzed (Fig 4B). BleachOto and BleachTyl exhibited high level of immunolabelling (Fig 4C and 4D). After 24 h only the Bleach group presented high immunolabelling (Fig 4E–4H). At 48 h (Fig 4I–4L), BleachOto and BleachTyl groups had lower immunolabelling for CGRP and Bleach presents moderate immunolabelling. The analysis according to time, it was observed that Bleach, BleachOto and BleachTyl group showed a decrease in marking of CGRP.

**Table 4:** Median scores assigned to CGRP immunolabeling in each coronal third for all groups studied.

		*Ctrl*			*Bleach*			*BleachOto*			*BleachTyl*		
Thirds		0h	24h	48h	0h	24h	48h	0h	24h	48h	0h	24h	48h
**Crown**	*Occlusal*	2Ab	2Ab	2Ab	5Aa	4ABa	3Ba	4Aab	3ABab	2Bb	4Aab	3ABab	2Bb
	*Medium*	2Ab	2Ab	2Aa	5Aa	4ABa	2Ba	4Aab	3ABab	2Ba	4Aab	3ABab	2Ba
	*Cervical*	2Ac	2Ab	2Aa	5Aa	4ABa	2Ba	3Abc	2Bb	2Ba	4Aab	2ABab	2Ba
**Root**	*Coronary*	2Ab	2Ab	2Aa	4Aa	3ABa	3Ba	3Ab	2Ab	2Aa	3Aab	2Aab	2Aa
	*Medium*	2Ab	2Aa	2Aa	4Aa	2Ba	2Ba	2Ab	2Aa	2Aa	2Ab	2Aa	2Aa
	*Apical*	2Aa	2Aa	2Aa	2Aa	2Aa	2Aa	2Aa	2Aa	2Aa	2Aa	2Aa	2Aa

* Means followed by different letters represent significant difference according to statistical analysis (p,0.05). Uppercase mean comparison between times within a group, and lowercase mean comparison between groups within a time (0h, 24h or 48h).

## Discussion

In the present study, the histopathological analysis of the pulp from rats subjected to bleaching treatment revealed a gradual and continuous improvement in the inflammatory tissue in the coronal portion. This fact was likely related to the recruitment of undifferentiated mesenchymal cells to the root pulp where tissue integrity was maintained, resulting in a more cellular tissue [25]. The tissue recovery of this experimental model occurs more vigorously than in humans. However, it is notable that this experimental model is able to mimic the conditions of human pulp tissue, with the presence of other cells in this tissue and intrapulpal pressure.

The Otosporin, was used on dental enamel immediately after the bleaching treatment. The histological analysis indicated greater amount of remaining cells for this group, with small areas of necrosis, whereas the group Bleach presented total necrosis of the coronal pulp. During bleaching, as well as at the time of Otosporin application, the periodontal tissues were protected with the resinous gingival barrier, which may have hampered the possible absorption of the drug by the periodontium. Based on the premise that the only difference between the BleachOto and Bleach groups was the application of Otosporin, it can be stated that the results observed in the BleachOto group show the real efficacy of this drug for inflammationtreatment and make further investigations possible. This drug reduce inflammatory infiltrate and promotes vasoconstriction of the inflamed area, reducing edema and discomfort, and stabilizing nerve cell membranes [26].

The paracetamol was selected because it is an anti-inflammatory with fewer side effects and with analgesic efficacy [27]. This drug acts directly on the arachidonic acid cascade, preventing the production of prostaglandins [27]. It was not possible to observe statistical difference, but the histological patterns of the BleachTyl group differed from those of the Bleach group. Because it acts systemically, this drug may have lost some of its effect on the pulp tissue. Further studies are needed to adjust the dosages of the bleaching products and drug.

The neuropeptides CGRP and SP found in pulp tissue are produced at the level of the cellular body and are carried in vesicles to the central nervous system and to the sensory receptors [28]. At the sensory receptor, such neuropeptides are released at a constant base level [29], which explains the presence of light immunolabeling in the control group. However, under pathological conditions, they are released in greater amounts at both the central and peripheral levels. It is therefore important to evaluate the presence of these neuropeptides in pulp tissue, because this can offer insights into the effects of bleaching agents and drugs on tooth sensitivity.

To evaluate the concentration of these neuropeptides, there are some specific methodologies such as PCR (polymerase chain reaction) that quantify the presence of these neuropeptides [30, 31]. However, this type of analysis does not provide us with localization results, which are important in this study. As a result, we chose immunohistochemical analysis for quantitative analysis because it reveals the quantity, presence, and localization of these neuropeptides.

Owing to the large areas of necrosis observed immediately after bleaching treatment in this study, immunolabeling of the neuropeptides was observed only in cellular precipitates from G2, which showed necrosis along the coronal pulp in all specimens. Positive immunolabeling of SP and CGRP was observed in the other groups, which indicates a painful sensation when compared to healthy dental pulps.

Tissue regeneration was observed within 24 h of bleaching, as was positive immunolabeling for both CGRP and SP in all groups that received bleaching treatment. However, there was a greater expression of the neuropeptides in the pulp of teeth that received bleaching treatment compared to that of the groups that did not undergo bleaching. This result agreed with that of a study by Caviedes-Bucheli, *et al*., in which both CGRP and SP were present in all pulps samples, but at increased levels in inflamed pulps [29].

The results of the present study suggested that the use of paracetamol and Otosporin positively affected the expression of pain-related neuropeptides, thereby minimizing the painful effects of this treatment. Otosporin suppresses inflammatory vascular changes to relieve the pressure induced by venous collapse, thereby decreasing nerve fiber stimulation and reducing the production of pain-related neuropeptides SP and CGRP. Souza, *et al*. [32], observed that treatment with corticosteroid-antibiotic combinations resulted in a slightly lower inflammatory reaction. With the reduction of the inflammatory process, consequently there will be a reduction in the levels of neuropeptides [32], confirming the results obtained in this study.

The effects of the drug paracetamol were most pronounced in the analysis of neuropeptide expression, owing to its predominant analgesic effect (inhibits COX-3) [27]. The indirect action of this drug in the production and release of these neuropeptides is driven by the reduction of the inflammatory response.

It is also worth noting that the control groups indicated that the drugs did not produce any adverse effects on healthy tissue. This fact indicated that the negative effects were caused only by the whitening gel. However, other authors state that the administration of anti-inflammatory substances can neither recover irreversible damages of the pulp [14, 33] nor protect the pulp tissue against adverse effects caused by professional tooth bleaching, but it was observed in this study that the anti-inflammatory effect of these drugs may contribute to reduce symptoms and restore health of the remaining pulp.

Despite the limitations of the experimental model (high concentrations of whitening product were used on the teeth of rats, which are inferior in hardness and show exacerbated biological response compared to human teeth), the results indicate that bleaching treatment can be highly damaging to the pulp tissue, however, when performed properly and using suitable dosages of the bleaching product, this treatment can be useful and safe.

## Conclusion

This study indicated that the topical application of Otosporin after bleaching treatment minimized inflammation and reduced the expression of pro-inflammatory neuropeptides. In addition, the administration of paracetamol also reduced inflammation, but did not influenced the expression of neuropeptides.

## Supporting information

S1 File Tables containing the raw data of this work. (DOCX)
