# Supplementary material for: Pulp response of rats submitted to bleaching and the use of different anti-inflammatory drugs
Source: PLoS One. 2019 Jan 8;14(1):e0210338. doi: 10.1371/journal.pone.0210338 (PMC6324791; doi:10.1371/journal.pone.0210338)
Supplement: S1 File — (DOCX) [file pone.0210338.s001.docx]

**Table 1** – Scores and medians assigned in the histological analysis in each coronary third for all control groups.

|  | | | | *Control* | | | | Otot | | | | Tyl | | | |
| --- | --- | --- | --- | --- | --- | --- | --- | --- | --- | --- | --- | --- | --- | --- | --- |
| Scores | 0h | | 24h | 48h | 0h | | 24h | 48h | 0h | | 24h | 48h |  |  |  |
| Crown | Occlusal third | 1 | **7/7** | | **7/7** | **7/7** | **7/7** | | **7/7** | **7/7** | **7/7** | | **7/7** | **7/7** |  |
|  |  | 2 | 0/7 | | 0/7 | 0/7 | 0/7 | | 0/7 | 0/7 | 0/7 | | 0/7 | 0/7 |  |
|  |  | 3 | 0/7 | | 0/7 | 0/7 | 0/7 | | 0/7 | 0/7 | 0/7 | | 0/7 | 0/7 |  |
|  |  | 4 | 0/7 | | 0/7 | 0/7 | 0/7 | | 0/7 | 0/7 | 0/7 | | 0/7 | 0/7 |  |
|  |  | 5 | 0/7 | | 0/7 | 0/7 | 0/7 | | 0/7 | 0/7 | 0/7 | | 0/7 | 0/7 |  |
|  |  | ***Mediana*** | **1** | | **1** | **1** | **1** | | **1** | **1** | **1** | | **1** | **1** |  |
|  | Middle third | 1 | **7/7** | | **7/7** | **7/7** | **7/7** | | **7/7** | **7/7** | **7/7** | | **7/7** | **7/7** |  |
|  |  | 2 | 0/7 | | 0/7 | 0/7 | 0/7 | | 0/7 | 0/7 | 0/7 | | 0/7 | 0/7 |  |
|  |  | 3 | 0/7 | | 0/7 | 0/7 | 0/7 | | 0/7 | 0/7 | 0/7 | | 0/7 | 0/7 |  |
|  |  | 4 | 0/7 | | 0/7 | 0/7 | 0/7 | | 0/7 | 0/7 | 0/7 | | 0/7 | 0/7 |  |
|  |  | 5 | 0/7 | | 0/7 | 0/7 | 0/7 | | 0/7 | 0/7 | 0/7 | | 0/7 | 0/7 |  |
|  |  | ***Medians*** | **1** | | **1** | **1** | **1** | | **1** | **1** | **1** | | **1** | **1** |  |
|  | Cervical third | 1 | **7/7** | | **7/7** | **7/7** | **7/7** | | **7/7** | **7/7** | **7/7** | | **7/7** | **7/7** |  |
|  |  | 2 | 0/7 | | 0/7 | 0/7 | 0/7 | | 0/7 | 0/7 | 0/7 | | 0/7 | 0/7 |  |
|  |  | 3 | 0/7 | | 0/7 | 0/7 | 0/7 | | 0/7 | 0/7 | 0/7 | | 0/7 | 0/7 |  |
|  |  | 4 | 0/7 | | 0/7 | 0/7 | 0/7 | | 0/7 | 0/7 | 0/7 | | 0/7 | 0/7 |  |
|  |  | 5 | 0/7 | | 0/7 | 0/7 | 0/7 | | 0/7 | 0/7 | 0/7 | | 0/7 | 0/7 |  |
|  |  | ***Medians*** | **1** | | **1** | **1** | **1** | | **1** | **1** | **1** | | **1** | **1** |  |
| Root | Coronary third | 1 | **7/7** | | **7/7** | **7/7** | **7/7** | | **7/7** | **7/7** | **7/7** | | **7/7** | **7/7** |  |
|  |  | 2 | 0/7 | | 0/7 | 0/7 | 0/7 | | 0/7 | 0/7 | 0/7 | | 0/7 | 0/7 |  |
|  |  | 3 | 0/7 | | 0/7 | 0/7 | 0/7 | | 0/7 | 0/7 | 0/7 | | 0/7 | 0/7 |  |
|  |  | 4 | 0/7 | | 0/7 | 0/7 | 0/7 | | 0/7 | 0/7 | 0/7 | | 0/7 | 0/7 |  |
|  |  | 5 | 0/7 | | 0/7 | 0/7 | 0/7 | | 0/7 | 0/7 | 0/7 | | 0/7 | 0/7 |  |
|  |  | ***Medians*** | **1** | | **1** | **1** | **1** | | **1** | **1** | **1** | | **1** | **1** |  |
|  | Middle third | 1 | **7/7** | | **7/7** | **7/7** | **7/7** | | **7/7** | **7/7** | **7/7** | | **7/7** | **7/7** |  |
|  |  | 2 | 0/7 | | 0/7 | 0/7 | 0/7 | | 0/7 | 0/7 | 0/7 | | 0/7 | 0/7 |  |
|  |  | 3 | 0/7 | | 0/7 | 0/7 | 0/7 | | 0/7 | 0/7 | 0/7 | | 0/7 | 0/7 |  |
|  |  | 4 | 0/7 | | 0/7 | 0/7 | 0/7 | | 0/7 | 0/7 | 0/7 | | 0/7 | 0/7 |  |
|  |  | 5 | 0/7 | | 0/7 | 0/7 | 0/7 | | 0/7 | 0/7 | 0/7 | | 0/7 | 0/7 |  |
|  |  | ***Medians*** | **1** | | **1** | **1** | **1** | | **1** | **1** | **1** | | **1** | **1** |  |
|  | Apical third | 1 | **7/7** | | **7/7** | **7/7** | **7/7** | | **7/7** | **7/7** | **7/7** | | **7/7** | **7/7** |  |
|  |  | 2 | 0/7 | | 0/7 | 0/7 | 0/7 | | 0/7 | 0/7 | 0/7 | | 0/7 | 0/7 |  |
|  |  | 3 | 0/7 | | 0/7 | 0/7 | 0/7 | | 0/7 | 0/7 | 0/7 | | 0/7 | 0/7 |  |
|  |  | 4 | 0/7 | | 0/7 | 0/7 | 0/7 | | 0/7 | 0/7 | 0/7 | | 0/7 | 0/7 |  |
|  |  | 5 | 0/7 | | 0/7 | 0/7 | 0/7 | | 0/7 | 0/7 | 0/7 | | 0/7 | 0/7 |  |
|  |  | ***Medians*** | **1** | | **1** | **1** | **1** | | **1** | **1** | **1** | | **1** | **1** |  |

**Table 2 –** Scores and medians assigned in the histological analysis in each coronary and radicular third for all bleached groups.

|  |  |  | *Control* | | | *Bleach* | | | *BleachOtot* | | | *BleachTyl* | | |
| --- | --- | --- | --- | --- | --- | --- | --- | --- | --- | --- | --- | --- | --- | --- |
|  |  | Scores | 0h | 24h | 48h | 0h | 24h | 48h | 0h | 24h | 48h | 0h | 24h | 48h |
| Crown | Occlusal third | 1 | **7/7** | **7/7** | **7/7** | 0/7 | 0/7 | 0/7 | 0/7 | 0/7 | 0/7 | 0/7 | 0/7 | 0/7 |
|  |  | 2 | 0/7 | 0/7 | 0/7 | 0/7 | 0/7 | 0/7 | 0/7 | 0/7 | 0/7 | 0/7 | 0/7 | 0/7 |
|  |  | 3 | 0/7 | 0/7 | 0/7 | 0/7 | **2/7** | **3/7** | **5/7** | **6/7** | **6/7** | 0/7 | **3/7** | **3/7** |
|  |  | 4 | 0/7 | 0/7 | 0/7 | 0/7 | **5/7** | **4/7** | **2/7** | **1/7** | **1/7** | **2/7** | **4/7** | **4/7** |
|  |  | 5 | 0/7 | 0/7 | 0/7 | **7/7** | 0/7 | 0/7 | 0/7 | 0/7 | 0/7 | **5/7** | 0/7 | 0/7 |
|  |  | ***Medians*** | **1** | **1** | **1** | **5** | **4** | **4** | **3** | **3** | **3** | **5** | **4** | **4** |
|  | Middle third | 1 | **7/7** | **7/7** | **7/7** | 0/7 | 0/7 | 0/7 | 0/7 | 0/7 | 0/7 | 0/7 | 0/7 | 0/7 |
|  |  | 2 | 0/7 | 0/7 | 0/7 | 0/7 | 0/7 | **3/7** | **3/7** | **4/7** | **5/7** | 0/7 | **1/7** | **2/7** |
|  |  | 3 | 0/7 | 0/7 | 0/7 | 0/7 | **6/7** | **4/7** | **2/7** | **3/7** | **2/7** | **2/7** | **6/7** | **5/7** |
|  |  | 4 | 0/7 | 0/7 | 0/7 | 0/7 | **1/7** | 0/7 | **2/7** | 0/7 | 0/7 | **3/7** | 0/7 | 0/7 |
|  |  | 5 | 0/7 | 0/7 | 0/7 | **7/7** | 0/7 | 0/7 | 0/7 | 0/7 | 0/7 | **2/7** | 0/7 | 0/7 |
|  |  | ***Medians*** | **1** | **1** | **1** | **5** | **3** | **3** | **2** | **2** | **2** | **4** | **3** | **3** |
|  | Cervical third | 1 | **7/7** | **7/7** | **7/7** | 0/7 | 0/7 | 0/7 | 0/7 | **3/7** | **3/7** | 0/7 | 0/7 | 0/7 |
|  |  | 2 | 0/7 | 0/7 | 0/7 | 0/7 | **5/7** | **6/7** | **4/7** | **4/7** | **4/7** | **1/7** | **4/7** | **5/7** |
|  |  | 3 | 0/7 | 0/7 | 0/7 | 0/7 | **2/7** | **1/7** | **2/7** | 0/7 | 0/7 | **1/7** | **3/7** | **2/7** |
|  |  | 4 | 0/7 | 0/7 | 0/7 | 0/7 | 0/7 | 0/7 | **1/7** | 0/7 | 0/7 | **5/7** | 0/7 | 0/7 |
|  |  | 5 | 0/7 | 0/7 | 0/7 | **7/7** | 0/7 | 0/7 | 0/7 | 0/7 | 0/7 | 0/7 | 0/7 | 0/7 |
|  |  | ***Medians*** | **1** | **1** | **1** | **5** | **2** | **2** | **2** | **2** | **2** | **4** | **2** | 2 |
| Root | Coronary third | 1 | **7/7** | **7/7** | **7/7** | 0/7 | **7/7** | **4/7** | 0/7 | **5/7** | **5/7** | 0/7 | **4/7** | **5/7** |
|  |  | 2 | 0/7 | 0/7 | 0/7 | **1/7** | 0/7 | **3/7** | **4/7** | **2/7** | **2/7** | **1/7** | **3/7** | **2/7** |
|  |  | 3 | 0/7 | 0/7 | 0/7 | **1/7** | 0/7 | 0/7 | **3/7** | 0/7 | 0/7 | **5/7** | 0/7 | 0/7 |
|  |  | 4 | 0/7 | 0/7 | 0/7 | **5/7** | 0/7 | 0/7 | 0/7 | 0/7 | 0/7 | **1/7** | 0/7 | 0/7 |
|  |  | 5 | 0/7 | 0/7 | 0/7 | 0/7 | 0/7 | 0/7 | 0/7 | 0/7 | 0/7 | 0/7 | 0/7 | 0/7 |
|  |  | ***Medians*** | **1** | **1** | **1** | **4** | **1** | **1** | **2** | **1** | **1** | **3** | **1** | **1** |
|  | Middle third | 1 | **7/7** | **7/7** | **7/7** | 0/7 | **6/7** | **7/7** | **5/7** | **7/7** | **7/7** | **1/7** | **7/7** | **7/7** |
|  |  | 2 | 0/7 | 0/7 | 0/7 | **5/7** | **1/7** | 0/7 | **2/7** | 0/7 | 0/7 | **5/7** | 0/7 | 0/7 |
|  |  | 3 | 0/7 | 0/7 | 0/7 | **2/7** | 0/7 | 0/7 | 0/7 | 0/7 | 0/7 | 1/7 | 0/7 | 0/7 |
|  |  | 4 | 0/7 | 0/7 | 0/7 | 0/7 | 0/7 | 0/7 | 0/7 | 0/7 | 0/7 | 0/7 | 0/7 | 0/7 |
|  |  | 5 | 0/7 | 0/7 | 0/7 | 0/7 | 0/7 | 0/7 | 0/7 | 0/7 | 0/7 | 0/7 | 0/7 | 0/7 |
|  |  | ***Medians*** | **1** | **1** | **1** | **2** | **1** | **1** | **1** | **1** | **1** | **2** | **1** | **1** |
|  | Apical third | 1 | **7/7** | **7/7** | **7/7** | **7/7** | **7/7** | **7/7** | **7/7** | **7/7** | **7/7** | **6/7** | **7/7** | **7/7** |
|  |  | 2 | 0/7 | 0/7 | 0/7 | 0/7 | 0/7 | 0/7 | 0/7 | 0/7 | 0/7 | 1/7 | 0/7 | 0/7 |
|  |  | 3 | 0/7 | 0/7 | 0/7 | 0/7 | 0/7 | 0/7 | 0/7 | 0/7 | 0/7 | 0/7 | 0/7 | 0/7 |
|  |  | 4 | 0/7 | 0/7 | 0/7 | 0/7 | 0/7 | 0/7 | 0/7 | 0/7 | 0/7 | 0/7 | 0/7 | 0/7 |
|  |  | 5 | 0/7 | 0/7 | 0/7 | 0/7 | 0/7 | 0/7 | 0/7 | 0/7 | 0/7 | 0/7 | 0/7 | 0/7 |
|  |  | ***Medians*** | **1** | **1** | **1** | **1** | **1** | **1** | **1** | **1** | **1** | **1** | **1** | **1** |

**Table 3** – Scores and medians attributed to SP immunostaining in each coronary and radicular third for all control groups.

|  | | | | *Control* | | | | Oto | | | | Tyl | | | |
| --- | --- | --- | --- | --- | --- | --- | --- | --- | --- | --- | --- | --- | --- | --- | --- |
| Scores | 0h | | 24h | 48h | 0h | | 24h | 48h | 0h | | 24h | 48h |  |  |  |
| Crown | Occlusal third | 1 | 0/7 | | 0/7 | 0/7 | 0/7 | | 0/7 | 0/7 | 0/7 | | 0/7 | 0/7 |  |
|  |  | 2 | **7/7** | | **7/7** | **7/7** | **7/7** | | **7/7** | **7/7** | **7/7** | | **7/7** | **7/7** |  |
|  |  | 3 | 0/7 | | 0/7 | 0/7 | 0/7 | | 0/7 | 0/7 | 0/7 | | 0/7 | 0/7 |  |
|  |  | 4 | 0/7 | | 0/7 | 0/7 | 0/7 | | 0/7 | 0/7 | 0/7 | | 0/7 | 0/7 |  |
|  |  | 5 | 0/7 | | 0/7 | 0/7 | 0/7 | | 0/7 | 0/7 | 0/7 | | 0/7 | 0/7 |  |
|  |  | ***Medians*** | **2** | | **2** | **2** | **2** | | **2** | **2** | **2** | | **2** | **2** |  |
|  | Middle third | 1 | 0/7 | | 0/7 | 0/7 | 0/7 | | 0/7 | 0/7 | 0/7 | | 0/7 | 0/7 |  |
|  |  | 2 | **7/7** | | **7/7** | **7/7** | **7/7** | | **7/7** | **7/7** | **7/7** | | **7/7** | **7/7** |  |
|  |  | 3 | 0/7 | | 0/7 | 0/7 | 0/7 | | 0/7 | 0/7 | 0/7 | | 0/7 | 0/7 |  |
|  |  | 4 | 0/7 | | 0/7 | 0/7 | 0/7 | | 0/7 | 0/7 | 0/7 | | 0/7 | 0/7 |  |
|  |  | 5 | 0/7 | | 0/7 | 0/7 | 0/7 | | 0/7 | 0/7 | 0/7 | | 0/7 | 0/7 |  |
|  |  | ***Medians*** | **2** | | **2** | **2** | **2** | | **2** | **2** | **2** | | **2** | **2** |  |
|  | Cervical third | 1 | 0/7 | | 0/7 | 0/7 | 0/7 | | 0/7 | 0/7 | 0/7 | | 0/7 | 0/7 |  |
|  |  | 2 | **7/7** | | **7/7** | **7/7** | **7/7** | | **7/7** | **7/7** | **7/7** | | **7/7** | **7/7** |  |
|  |  | 3 | 0/7 | | 0/7 | 0/7 | 0/7 | | 0/7 | 0/7 | 0/7 | | 0/7 | 0/7 |  |
|  |  | 4 | 0/7 | | 0/7 | 0/7 | 0/7 | | 0/7 | 0/7 | 0/7 | | 0/7 | 0/7 |  |
|  |  | 5 | 0/7 | | 0/7 | 0/7 | 0/7 | | 0/7 | 0/7 | 0/7 | | 0/7 | 0/7 |  |
|  |  | ***Medians*** | **2** | | **2** | **2** | **2** | | **2** | **2** | **2** | | **2** | **2** |  |
| Root | Coronary third | 1 | 0/7 | | 0/7 | 0/7 | 0/7 | | 0/7 | 0/7 | 0/7 | | 0/7 | 0/7 |  |
|  |  | 2 | **7/7** | | **7/7** | **7/7** | **7/7** | | **7/7** | **7/7** | **7/7** | | **7/7** | **7/7** |  |
|  |  | 3 | 0/7 | | 0/7 | 0/7 | 0/7 | | 0/7 | 0/7 | 0/7 | | 0/7 | 0/7 |  |
|  |  | 4 | 0/7 | | 0/7 | 0/7 | 0/7 | | 0/7 | 0/7 | 0/7 | | 0/7 | 0/7 |  |
|  |  | 5 | 0/7 | | 0/7 | 0/7 | 0/7 | | 0/7 | 0/7 | 0/7 | | 0/7 | 0/7 |  |
|  |  | ***Medians*** | **2** | | **2** | **2** | **2** | | **2** | **2** | **2** | | **2** | **2** |  |
|  | Middle third | 1 | 0/7 | | 0/7 | 0/7 | 0/7 | | 0/7 | 0/7 | 0/7 | | 0/7 | 0/7 |  |
|  |  | 2 | **7/7** | | **7/7** | **7/7** | **7/7** | | **7/7** | **7/7** | **7/7** | | **7/7** | **7/7** |  |
|  |  | 3 | 0/7 | | 0/7 | 0/7 | 0/7 | | 0/7 | 0/7 | 0/7 | | 0/7 | 0/7 |  |
|  |  | 4 | 0/7 | | 0/7 | 0/7 | 0/7 | | 0/7 | 0/7 | 0/7 | | 0/7 | 0/7 |  |
|  |  | 5 | 0/7 | | 0/7 | 0/7 | 0/7 | | 0/7 | 0/7 | 0/7 | | 0/7 | 0/7 |  |
|  |  | ***Medians*** | **2** | | **2** | **2** | **2** | | **2** | **2** | **2** | | **2** | **2** |  |
|  | Apical third | 1 | 0/7 | | 0/7 | 0/7 | 0/7 | | 0/7 | 0/7 | 0/7 | | 0/7 | 0/7 |  |
|  |  | 2 | **7/7** | | **7/7** | **7/7** | **7/7** | | **7/7** | **7/7** | **7/7** | | **7/7** | **7/7** |  |
|  |  | 3 | 0/7 | | 0/7 | 0/7 | 0/7 | | 0/7 | 0/7 | 0/7 | | 0/7 | 0/7 |  |
|  |  | 4 | 0/7 | | 0/7 | 0/7 | 0/7 | | 0/7 | 0/7 | 0/7 | | 0/7 | 0/7 |  |
|  |  | 5 | 0/7 | | 0/7 | 0/7 | 0/7 | | 0/7 | 0/7 | 0/7 | | 0/7 | 0/7 |  |
|  |  | ***Medians*** | **2** | | **2** | **2** | **2** | | **2** | **2** | **2** | | **2** | **2** |  |

**Table 4** – Scores attributed to CGRP immunoblotting in each coronary and radicular third for all control groups.

|  | | | | *Control* | | | | Oto | | | | Tyl | | | |
| --- | --- | --- | --- | --- | --- | --- | --- | --- | --- | --- | --- | --- | --- | --- | --- |
| Scores | 0h | | 24h | 48h | 0h | | 24h | 48h | 0h | | 24h | 48h |  |  |  |
| Crown | Occlusal third | 1 | 0/7 | | 0/7 | 0/7 | 0/7 | | 0/7 | 0/7 | 0/7 | | 0/7 | 0/7 |  |
|  |  | 2 | **7/7** | | **7/7** | **7/7** | **7/7** | | **7/7** | **7/7** | **7/7** | | **7/7** | **7/7** |  |
|  |  | 3 | 0/7 | | 0/7 | 0/7 | 0/7 | | 0/7 | 0/7 | 0/7 | | 0/7 | 0/7 |  |
|  |  | 4 | 0/7 | | 0/7 | 0/7 | 0/7 | | 0/7 | 0/7 | 0/7 | | 0/7 | 0/7 |  |
|  |  | 5 | 0/7 | | 0/7 | 0/7 | 0/7 | | 0/7 | 0/7 | 0/7 | | 0/7 | 0/7 |  |
|  |  | ***Medians*** | **2** | | **2** | **2** | **2** | | **2** | **2** | **2** | | **2** | **2** |  |
|  | Middle third | 1 | 0/7 | | 0/7 | 0/7 | 0/7 | | 0/7 | 0/7 | 0/7 | | 0/7 | 0/7 |  |
|  |  | 2 | **7/7** | | **7/7** | **7/7** | **7/7** | | **7/7** | **7/7** | **7/7** | | **7/7** | **7/7** |  |
|  |  | 3 | 0/7 | | 0/7 | 0/7 | 0/7 | | 0/7 | 0/7 | 0/7 | | 0/7 | 0/7 |  |
|  |  | 4 | 0/7 | | 0/7 | 0/7 | 0/7 | | 0/7 | 0/7 | 0/7 | | 0/7 | 0/7 |  |
|  |  | 5 | 0/7 | | 0/7 | 0/7 | 0/7 | | 0/7 | 0/7 | 0/7 | | 0/7 | 0/7 |  |
|  |  | ***Medians*** | **2** | | **2** | **2** | **2** | | **2** | **2** | **2** | | **2** | **2** |  |
|  | Cervical third | 1 | 0/7 | | 0/7 | 0/7 | 0/7 | | 0/7 | 0/7 | 0/7 | | 0/7 | 0/7 |  |
|  |  | 2 | **7/7** | | **7/7** | **7/7** | **7/7** | | **7/7** | **7/7** | **7/7** | | **7/7** | **7/7** |  |
|  |  | 3 | 0/7 | | 0/7 | 0/7 | 0/7 | | 0/7 | 0/7 | 0/7 | | 0/7 | 0/7 |  |
|  |  | 4 | 0/7 | | 0/7 | 0/7 | 0/7 | | 0/7 | 0/7 | 0/7 | | 0/7 | 0/7 |  |
|  |  | 5 | 0/7 | | 0/7 | 0/7 | 0/7 | | 0/7 | 0/7 | 0/7 | | 0/7 | 0/7 |  |
|  |  | ***Medians*** | **2** | | **2** | **2** | **2** | | **2** | **2** | **2** | | **2** | **2** |  |
| Root | Coronary third | 1 | 0/7 | | 0/7 | 0/7 | 0/7 | | 0/7 | 0/7 | 0/7 | | 0/7 | 0/7 |  |
|  |  | 2 | **7/7** | | **7/7** | **7/7** | **7/7** | | **7/7** | **7/7** | **7/7** | | **7/7** | **7/7** |  |
|  |  | 3 | 0/7 | | 0/7 | 0/7 | 0/7 | | 0/7 | 0/7 | 0/7 | | 0/7 | 0/7 |  |
|  |  | 4 | 0/7 | | 0/7 | 0/7 | 0/7 | | 0/7 | 0/7 | 0/7 | | 0/7 | 0/7 |  |
|  |  | 5 | 0/7 | | 0/7 | 0/7 | 0/7 | | 0/7 | 0/7 | 0/7 | | 0/7 | 0/7 |  |
|  |  | ***Medians*** | **2** | | **2** | **2** | **2** | | **2** | **2** | **2** | | **2** | **2** |  |
|  | Middle third | 1 | 0/7 | | 0/7 | 0/7 | 0/7 | | 0/7 | 0/7 | 0/7 | | 0/7 | 0/7 |  |
|  |  | 2 | **7/7** | | **7/7** | **7/7** | **7/7** | | **7/7** | **7/7** | **7/7** | | **7/7** | **7/7** |  |
|  |  | 3 | 0/7 | | 0/7 | 0/7 | 0/7 | | 0/7 | 0/7 | 0/7 | | 0/7 | 0/7 |  |
|  |  | 4 | 0/7 | | 0/7 | 0/7 | 0/7 | | 0/7 | 0/7 | 0/7 | | 0/7 | 0/7 |  |
|  |  | 5 | 0/7 | | 0/7 | 0/7 | 0/7 | | 0/7 | 0/7 | 0/7 | | 0/7 | 0/7 |  |
|  |  | ***Medians*** | **2** | | **2** | **2** | **2** | | **2** | **2** | **2** | | **2** | **2** |  |
|  | Apical third | 1 | 0/7 | | 0/7 | 0/7 | 0/7 | | 0/7 | 0/7 | 0/7 | | 0/7 | 0/7 |  |
|  |  | 2 | **7/7** | | **7/7** | **7/7** | **7/7** | | **7/7** | **7/7** | **7/7** | | **7/7** | **7/7** |  |
|  |  | 3 | 0/7 | | 0/7 | 0/7 | 0/7 | | 0/7 | 0/7 | 0/7 | | 0/7 | 0/7 |  |
|  |  | 4 | 0/7 | | 0/7 | 0/7 | 0/7 | | 0/7 | 0/7 | 0/7 | | 0/7 | 0/7 |  |
|  |  | 5 | 0/7 | | 0/7 | 0/7 | 0/7 | | 0/7 | 0/7 | 0/7 | | 0/7 | 0/7 |  |
|  |  | ***Medians*** | **2** | | **2** | **2** | **2** | | **2** | **2** | **2** | | **2** | **2** |  |

**Table 5** – Scores attributed to SP immunostaining in each coronary and radicular third for all bleached groups.

|  |  |  | *Control* | | | *Bleach* | | | *BleachOtot* | | | *BleachTyl* | | |
| --- | --- | --- | --- | --- | --- | --- | --- | --- | --- | --- | --- | --- | --- | --- |
|  |  | Scores | 0h | 24h | 48h | 0h | 24h | 48h | 0h | 24h | 48h | 0h | 24h | 48h |
| Crown | Occlusal third | 1 | 0/7 | 0/7 | 0/7 | 0/7 | 0/7 | 0/7 | 0/7 | 0/7 | 0/7 | 0/7 | 0/7 | 0/7 |
|  |  | 2 | **7/7** | **7/7** | **7/7** | 07 | 0/7 | 0/7 | 0/7 | **3/7** | **7/7** | 0/7 | 0/7 | **7/7** |
|  |  | 3 | 0/7 | 0/7 | 0/7 | 0/7 | 0/7 | **7/7** | 0/7 | **4/7** | 0/7 | 0/7 | **7/7** | 0/7 |
|  |  | 4 | 0/7 | 0/7 | 0/7 | 0/7 | **7/7** | 0/7 | **4/7** | 0/7 | 0/7 | **3/7** | 0/7 | 0/7 |
|  |  | 5 | 0/7 | 0/7 | 0/7 | **7/7** | 0/7 | 0/7 | **3/7** | 0/7 | 0/7 | **4/7** | 0/7 | 0/7 |
|  |  | ***Medians*** | **2** | **2** | **2** | **5** | **4** | **3** | **4** | **3** | **2** | **5** | **3** | **2** |
|  | Middle third | 1 | 0/7 | 0/7 | 0/7 | 0/7 | 0/7 | 0/7 | 0/7 | 0/7 | 0/7 | 0/7 | 0/7 | 0/7 |
|  |  | 2 | **7/7** | **7/7** | **7/7** | 0/7 | 0/7 | **4/7** | 0/7 | **7/7** | **7/7** | 0/7 | **3/7** | **7/7** |
|  |  | 3 | 0/7 | 0/7 | 0/7 | 0/7 | **3/7** | **3/7** | **1/7** | 0/7 | 0/7 | 0/7 | **4/7** | 0/7 |
|  |  | 4 | 0/7 | 0/7 | 0/7 | 0/7 | **4/7** | 0/7 | **6/7** | 0/7 | 0/7 | **4/7** | 0/7 | 0/7 |
|  |  | 5 | 0/7 | 0/7 | 0/7 | **7/7** | 0/7 | 0/7 | 0/7 | 0/7 | 0/7 | **3/7** | 0/7 | 0/7 |
|  |  | ***Medians*** | **2** | **2** | **2** | **5** | **4** | **2** | **4** | **2** | **2** | **4** | **3** | **2** |
|  | Cervical third | 1 | 0/7 | 0/7 | 0/7 | 0/7 | 0/7 | 0/7 | 0/7 | 0/7 | 0/7 | 0/7 | 0/7 | 0/7 |
|  |  | 2 | **7/7** | **7/7** | **7/7** | 0/7 | 0/7 | **7/7** | 0/7 | **7/7** | **7/7** | 0/7 | **7/7** | **7/7** |
|  |  | 3 | 0/7 | 0/7 | 0/7 | 0/7 | **7/7** | 0/7 | **7/7** | 0/7 | 0/7 | 0/7 | 0/7 | 0/7 |
|  |  | 4 | 0/7 | 0/7 | 0/7 | 0/7 | 0/7 | 0/7 | 0/7 | 0/7 | 0/7 | **7/7** | 0/7 | 0/7 |
|  |  | 5 | 0/7 | 0/7 | 0/7 | **7/7** | 0/7 | 0/7 | 0/7 | 0/7 | 0/7 | 0/7 | 0/7 | 0/7 |
|  |  | ***Medians*** | **2** | **2** | **2** | **5** | **3** | **2** | **3** | **2** | **2** | **4** | **2** | **2** |
| Root | Coronary third | 1 | 0/7 | 0/7 | 0/7 | 0/7 | 0/7 | 0/7 | 0/7 | 0/7 | 0/7 | 0/7 | 0/7 | 0/7 |
|  |  | 2 | **7/7** | **7/7** | **7/7** | 0/7 | 0/7 | 0/7 | 0/7 | **7/7** | **7/7** | **7/7** | **7/7** | **7/7** |
|  |  | 3 | 0/7 | 0/7 | 0/7 | **2/7** | **7/7** | **7/7** | **7/7** | 0/7 | 0/7 | 0/7 | 0/7 | 0/7 |
|  |  | 4 | 0/7 | 0/7 | 0/7 | **5/7** | 0/7 | 0/7 | 0/7 | 0/7 | 0/7 | 0/7 | 0/7 | 0/7 |
|  |  | 5 | 0/7 | 0/7 | 0/7 | 0/7 | 0/7 | 0/7 | 0/7 | 0/7 | 0/7 | 0/7 | 0/7 | 0/7 |
|  |  | ***Medians*** | **2** | **2** | **2** | **4** | **3** | **3** | **3** | **2** | **2** | **2** | **2** | **2** |
|  | Middle third | 1 | 0/7 | 0/7 | 0/7 | 0/7 | 0/7 | 0/7 | 0/7 | 0/7 | 0/7 | 0/7 | 0/7 | 0/7 |
|  |  | 2 | **7/7** | **7/7** | **7/7** | 0/7 | **7/7** | **7/7** | **7/7** | **7/7** | **7/7** | **7/7** | **7/7** | **7/7** |
|  |  | 3 | 0/7 | 0/7 | 0/7 | **3/7** | 0/7 | 0/7 | 0/7 | 0/7 | 0/7 | 0/7 | 0/7 | 0/7 |
|  |  | 4 | 0/7 | 0/7 | 0/7 | **4/7** | 0/7 | 0/7 | 0/7 | 0/7 | 0/7 | 0/7 | 0/7 | 0/7 |
|  |  | 5 | 0/7 | 0/7 | 0/7 | 0/7 | 0/7 | 0/7 | 0/7 | 0/7 | 0/7 | 0/7 | 0/7 | 0/7 |
|  |  | ***Medians*** | **2** | **2** | **2** | **4** | **2** | **2** | **2** | **2** | **2** | **2** | **2** | **2** |
|  | Apical third | 1 | 0/7 | 0/7 | 0/7 | 0/7 | 0/7 | 0/7 | 0/7 | 0/7 | 0/7 | 0/7 | 0/7 | 0/7 |
|  |  | 2 | **7/7** | **7/7** | **7/7** | **77** | **5/7** | **7/7** | **4/7** | **4/7** | **7/7** | **5/7** | **7/7** | **7/7** |
|  |  | 3 | 0/7 | 0/7 | 0/7 | 0/7 | **2/7** | 0/7 | **3/7** | **3/7** | 0/7 | **3/7** | 0/7 | 0/7 |
|  |  | 4 | 0/7 | 0/7 | 0/7 | 0/7 | 0/7 | 0/7 | 0/7 | 0/7 | 0/7 | 0/7 | 0/7 | 0/7 |
|  |  | 5 | 0/7 | 0/7 | 0/7 | 0/7 | 0/7 | 0/7 | 0/7 | 0/7 | 0/7 | 0/7 | 0/7 | 0/7 |
|  |  | ***Medians*** | **2** | **2** | **2** | **2** | **2** | **2** | **2** | **2** | **2** | **2** | **2** | **2** |

**Table 6** – Scores attributed to CGRP immunostaining in each coronary and radicular third for all bleached groups.

|  |  |  | *Control* | | | *Bleach* | | | *BleachOtot* | | | *BleachTyl* | | |
| --- | --- | --- | --- | --- | --- | --- | --- | --- | --- | --- | --- | --- | --- | --- |
|  |  | Scores | 0h | 24h | 48h | 0h | 24h | 48h | 0h | 24h | 48h | 0h | 24h | 48h |
| Crown | Occlusal third | 1 | 0/7 | 0/7 | 0/7 | 0/7 | 0/7 | 0/7 | 0/7 | 0/7 | 0/7 | 0/7 | 0/7 | 0/7 |
|  |  | 2 | **7/7** | **7/7** | **7/7** | 07 | 0/7 | 0/7 | 0/7 | 0/7 | **7/7** | 0/7 | 0/7 | **7/7** |
|  |  | 3 | 0/7 | 0/7 | 0/7 | 0/7 | 0/7 | **7/7** | 0/7 | **7/7** | 0/7 | 0/7 | **7/7** | 0/7 |
|  |  | 4 | 0/7 | 0/7 | 0/7 | 0/7 | **7/7** | 0/7 | **4/7** | 0/7 | 0/7 | **4/7** | 0/7 | 0/7 |
|  |  | 5 | 0/7 | 0/7 | 0/7 | **7/7** | 0/7 | 0/7 | **3/7** | 0/7 | 0/7 | **3/7** | 0/7 | 0/7 |
|  |  | ***Medians*** | **2** | **2** | **2** | **5** | **4** | **3** | **4** | **3** | **2** | **4** | **3** | **2** |
|  | Middle third | 1 | 0/7 | 0/7 | 0/7 | 0/7 | 0/7 | 0/7 | 0/7 | 0/7 | 0/7 | 0/7 | 0/7 | 0/7 |
|  |  | 2 | **7/7** | **7/7** | **7/7** | 0/7 | 0/7 | **4/7** | 0/7 | **3/7** | **7/7** | 0/7 | 0/7 | **7/7** |
|  |  | 3 | 0/7 | 0/7 | 0/7 | 0/7 | 0/7 | **3/7** | **1/7** | **4/7** | 0/7 | 0/7 | **7/7** | 0/7 |
|  |  | 4 | 0/7 | 0/7 | 0/7 | 0/7 | **7/7** | 0/7 | **6/7** | 0/7 | 0/7 | **4/7** | 0/7 | 0/7 |
|  |  | 5 | 0/7 | 0/7 | 0/7 | **7/7** | 0/7 | 0/7 | 0/7 | 0/7 | 0/7 | **3/7** | 0/7 | 0/7 |
|  |  | ***Medians*** | **2** | **2** | **2** | **5** | **4** | **2** | **4** | **3** | **2** | **4** | **3** | **2** |
|  | Cervical third | 1 | 0/7 | 0/7 | 0/7 | 0/7 | 0/7 | 0/7 | 0/7 | 0/7 | 0/7 | 0/7 | 0/7 | 0/7 |
|  |  | 2 | **7/7** | **7/7** | **7/7** | 0/7 | 0/7 | **7/7** | 0/7 | **7/7** | **7/7** | 0/7 | **4/7** | **7/7** |
|  |  | 3 | 0/7 | 0/7 | 0/7 | 0/7 | **3/7** | 0/7 | **7/7** | 0/7 | 0/7 | 0/7 | **3/7** | 0/7 |
|  |  | 4 | 0/7 | 0/7 | 0/7 | 0/7 | **4/7** | 0/7 | 0/7 | 0/7 | 0/7 | **7/7** | 0/7 | 0/7 |
|  |  | 5 | 0/7 | 0/7 | 0/7 | **7/7** | 0/7 | 0/7 | 0/7 | 0/7 | 0/7 | 0/7 | 0/7 | 0/7 |
|  |  | ***Medians*** | **2** | **2** | **2** | **5** | **4** | **2** | **3** | **2** | **2** | **4** | **2** | **2** |
| Root | Coronary third | 1 | 0/7 | 0/7 | 0/7 | 0/7 | 0/7 | 0/7 | 0/7 | 0/7 | 0/7 | 0/7 | 0/7 | 0/7 |
|  |  | 2 | **7/7** | **7/7** | **7/7** | 0/7 | 0/7 | 0/7 | 0/7 | **7/7** | **7/7** | **1/7** | **7/7** | **7/7** |
|  |  | 3 | 0/7 | 0/7 | 0/7 | **2/7** | **7/7** | **7/7** | **7/7** | 0/7 | 0/7 | **6/7** | 0/7 | 0/7 |
|  |  | 4 | 0/7 | 0/7 | 0/7 | **5/7** | 0/7 | 0/7 | 0/7 | 0/7 | 0/7 | 0/7 | 0/7 | 0/7 |
|  |  | 5 | 0/7 | 0/7 | 0/7 | 0/7 | 0/7 | 0/7 | 0/7 | 0/7 | 0/7 | 0/7 | 0/7 | 0/7 |
|  |  | ***Medians*** | **2** | **2** | **2** | **4** | **3** | **3** | **3** | **2** | **2** | **3** | **2** | **2** |
|  | Middle third | 1 | 0/7 | 0/7 | 0/7 | 0/7 | 0/7 | 0/7 | 0/7 | 0/7 | 0/7 | 0/7 | 0/7 | 0/7 |
|  |  | 2 | **7/7** | **7/7** | **7/7** | 0/7 | **7/7** | **7/7** | **7/7** | **7/7** | **7/7** | **7/7** | **7/7** | **7/7** |
|  |  | 3 | 0/7 | 0/7 | 0/7 | **3/7** | 0/7 | 0/7 | 0/7 | 0/7 | 0/7 | 0/7 | 0/7 | 0/7 |
|  |  | 4 | 0/7 | 0/7 | 0/7 | **4/7** | 0/7 | 0/7 | 0/7 | 0/7 | 0/7 | 0/7 | 0/7 | 0/7 |
|  |  | 5 | 0/7 | 0/7 | 0/7 | 0/7 | 0/7 | 0/7 | 0/7 | 0/7 | 0/7 | 0/7 | 0/7 | 0/7 |
|  |  | ***Medians*** | **2** | **2** | **2** | **4** | **2** | **2** | **2** | **2** | **2** | **2** | **2** | **2** |
|  | Apical third | 1 | 0/7 | 0/7 | 0/7 | 0/7 | 0/7 | 0/7 | 0/7 | 0/7 | 0/7 | 0/7 | 0/7 | 0/7 |
|  |  | 2 | **7/7** | **7/7** | **7/7** | **7/7** | **7/7** | **7/7** | **4/7** | **7/7** | **7/7** | **4/7** | **7/7** | **7/7** |
|  |  | 3 | 0/7 | 0/7 | 0/7 | 0/7 | 0/7 | 0/7 | **3/7** | 0/7 | 0/7 | **3/7** | 0/7 | 0/7 |
|  |  | 4 | 0/7 | 0/7 | 0/7 | 0/7 | 0/7 | 0/7 | 0/7 | 0/7 | 0/7 | 0/7 | 0/7 | 0/7 |
|  |  | 5 | 0/7 | 0/7 | 0/7 | 0/7 | 0/7 | 0/7 | 0/7 | 0/7 | 0/7 | 0/7 | 0/7 | 0/7 |
|  |  | ***Medians*** | **2** | **2** | **2** | **2** | **2** | **2** | **2** | **2** | **2** | **2** | **2** | **2** |
